# Comparison of anterior sclera thickness in emmetropes and myopes

**DOI:** 10.1186/s12886-023-02775-x

**Published:** 2023-02-14

**Authors:** Jie Zhou, Hai He, Qiang Yang, Jiang-Ying Wang, Zhi-Peng You, Li-Li Liu

**Affiliations:** Department of Ophthalmology, Affiliated Eye Hospital of Nanchang University, No.463 of Bayi Road, Donghu District, Nanchang, Jiangxi 330006 China

**Keywords:** Myopia, Axial length, Anterior scleral thickness, Optical coherence tomography

## Abstract

**Background:**

This study aimed to compare anterior scleral thicknesses (ASTs) in people with emmetropia and myopia to explore the effect of myopia on AST.

**Methods:**

In this cross-sectional study, 93 participants (i.e., 93 eyes) with emmetropia and myopia underwent ocular imaging via anterior segment optical coherence tomography. We acquired raw B-scan OCT images along each of the four meridians (superior, inferior, nasal, and temporal), The AST was estimated from the limbus to a distance of 6 mm. The participants were aged between 20 and 50 years (mean age: 30.2 ± 8.8 years). The axial length (AL) was 22.50 ~ 33.04 mm (mean AL: 26.51 ± 2.65 mm), and the spherical equivalent (SE) was + 0.50 ~ 27.5 D (mean SE: −7.20 ± 6.5 D). The selected sample comprised 37 males and 56 females who were categorized as emmetropes, mild–moderate myopes, or high myopes. The four meridians of AST, AL, and refractive error were observed.

**Results:**

The AL was significantly negatively correlated with the four meridians of AST (the *r* value ranged between − 0.511 and − 0.228, *P <* 0.05). There was no significant correlation between age and inferior diameter (*r* = 0.113, *P* = 0.314), but age was positively correlated with the average AST of the superior, temporal, and nasal diameters (the *r* value ranged between 0.452 and 0.552, *P* < 0.05). There was no significant correlation between sex and AST (the *T* value ranged between − 1.816 and − 0.130, *P* > 0.05). Except for the inferior diameters of 1 mm, 5 mm, and 6 mm and the temporal diameter of 1 mm, the four diameters in the emmetropia group and the high myopia group were statistically significant at a distance of 0 ~ 6 mm from the limbus (*P* < 0.05).

**Conclusion:**

The AST is negatively correlated with AL and positively correlated with age. Compared with emmetropic eyes, the AST is thinner in highly myopic eyes. Myopia affects AST, which may be useful for monitoring progression in cases of myopia.

## Background

Myopia has become a global public health problem [1]. Extensive evidence has shown that myopic axial elongation is related to the acceleration of connective tissue remodeling; this process exerts mechanical tensile force on the eyeball wall, causing retinal and choroidal damage and biomechanical changes in the sclera. These biomechanical changes play an important role in the occurrence and development of myopia.

There exists a common cross-species emmetropization mechanism that matches the axial length (AL) of the eye with its optical system to regulate scleral remodeling and axial elongation [2–5]. Many studies have confirmed the changes in scleral composition, structure, biomechanics, and remodeling during the progression of myopia [6, 7]. However, there are few reports on the connection between myopia and the anterior sclera. Although several foreign studies on the anterior sclera in myopia have been published, the results are controversial [8–11]. The study of the anterior sclera was quantified using optical coherence tomography (OCT) of the anterior segment (AS) (AS-OCT), which makes it possible to observe and measure the changes in the anterior sclera. Therefore, this study investigated the relationship between anterior scleral thickness (AST) and age, sex, AL, degree of myopia, and mean corneal curvature (Km). The results will help clarify the correlation between AST and myopia and provide a theoretical basis for the prevention and control of the disease.

## Methods

The participants were classified into the following three categories based on SE refractive error (defined as spherical power plus one-half of the cylindrical power): emmetropes (+ 0.5 to − 0.25 D, *n* = 23), mild and moderate myopes (–0.5 to − 6.00 D, *n* = 32), and high myopes ( ≤ − 6.25 D, *n* = 38). This was to investigate the relationship between the increasing degree of myopia and AST. As shown in Table 1, these groups did not differ significantly in age or sex (Table 1).

**Table 1:** Basic information of the three groups

Groups	Age	AL	Female (%)	SE	n
Emmetropes	29.9 ± 8.1	23.5 ± 0.8	56.50%	-0.1 ± 0.20	23
Mild-moderates	28.1 ± 7.6	25.0 ± 0.5	62.50%	-4.9 ± 1.45	32
High myopes	32.2 ± 9.9	29.05 ± 2.0	60.52%	-13.3 ± 5.67	38

Mean ± SD demographic and ocular characteristics of the study participants, categorized according to refractive group

*SE* denotes spherical equivalent, *AL* denotes axial length

This study was approved by the Medical Ethics Committee of Nanchang University, Jiangxi, China and adhered to the principles of the Declaration of Helsinki. The nature of the study was explained to all participants, and appropriate consent, both written and verbal, was obtained.

The inclusion criteria were as follows: 1) AL ≥ 22.5 mm or SE ≤ + 0.5 D. (2) Aged between 20 and 50 years. (3) If both eyes of a participant met all the inclusion and exclusion criteria, only the right eye was chosen for the study. None of the participants wore rigid contact lenses, and those who wore soft contact lenses abstained from wearing them for 1 week before their participation in the study.

The exclusion criteria were as follows: (1) Presence of ophthalmopathy except for myopia (e.g., high myopia choroidal neovascularization, pterygium, retinal vein occlusion, glaucoma, uveitis, history of ocular trauma, rheumatoid arthritis and other auto-inflammatory); (2) Using systemic or topical medication; (3) Wearing or discontinuation of keratoplasty lenses within 1 week; (4) Anisometropia of ≥ 1.5 D and Km < 46.0 D; (5) History of ocular surgery (e.g., retinal laser photocoagulation, vitreous injection, and cataract surgery); (6) Presence of ocular hypertension; (7) Non-cooperating patients or those who could not complete the ophthalmic examination; (8) Presence of chronic diseases, such as systemic hypertension and diabetes mellitus; (9) Poor-quality AS-OCT scan results.

All the participants underwent an initial comprehensive eye examination; this included slit-lamp biomicroscopy and a dilated fundus examination, a refractive error assessment (KR-8900 autorefractor, Topcon, Japan), measurements of best-corrected visual acuity and intraocular pressure (Full Auto Tonometer TX-F, Topcon, Japan). Scleral thickness was assessed (CIRRUS HD-OCT5000, Carl Zeiss Meditec, U.S.A.), AL and mean meridian keratometry were measured (IOL Master 5.5, Carl Zeiss Meditec AG, Germany), central refraction was determined (ARK-510 autorefractor, Nidek, Japan), and wide-field fundus images were captured (Scanning Laser Ophthalmoscope, Daytona P200T, Optos, U.K.).

An average of five consecutive measurements were obtained for the AL, an average of three consecutive measurements were obtained for the Km, and an average of three measurements were obtained for the central refraction. All measurements were obtained by the same examiner. Subjective refraction was performed on all participants by a trained optometrist. The SE was calculated as the sphere plus half a cylinder. An Scanning Laser Ophthalmoscope test was used to further exclude fundus diseases other than myopia.

### Measurement of anterior scleral thickness

**Fig. 1:**
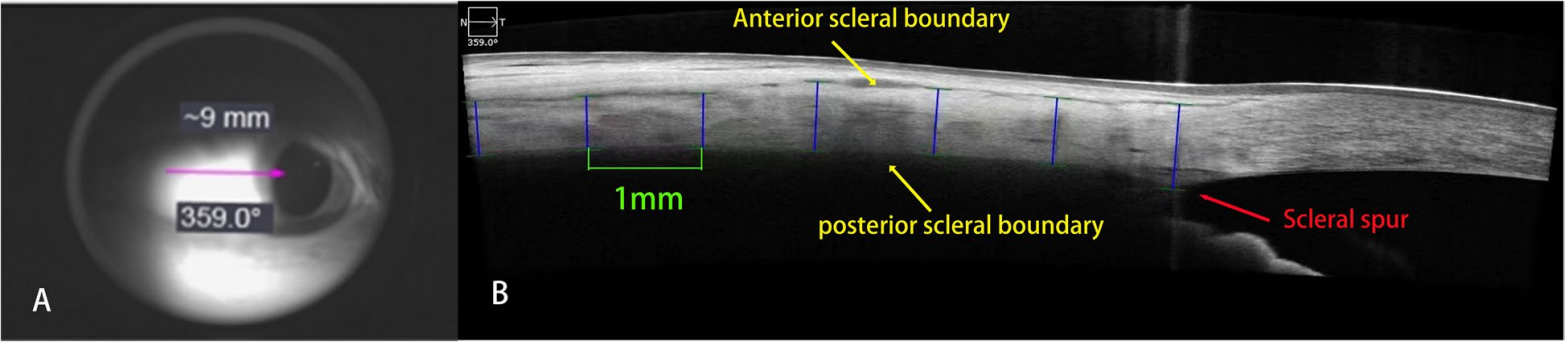
**A** Single-line scan passing through the temporal scleral reflex. **B** Cropped raw B-scan image of the anterior sclera (dimension of the exported image: length: 9 mm, depth: 2.6 mm). The yellow arrowheads indicate the anterior scleral boundary and posterior scleral boundary, and the red arrowhead indicates the location of the scleral spur (reference point). Segmented optical coherence tomography image obtained after analysis with custom-designed software. The solid blue lines denote the 1-mm intervals at which the scleral thickness was measured

An anterior segment module was attached in front of the objective lens of the HD-OCT instrument. A 9-mm single-line scan protocol (length: 9 mm, depth: 2.6 mm) was used to acquire raw B-scan OCT images along each of the four meridians (superior, inferior, temporal, and nasal) while the participant fixated an appropriately located target; an image of the sclera was acquired within this target. During imaging, the scan line was positioned such that it passed above the scleral reflex in each gaze position to ensure that the AST results were obtained consistently in the same region of interest in all the participants (Fig. 1).

Scleral thickness was determined by manual measurement using caliper software included in the instrument. Only the images of the right eyes were used to analyze AST. We acquired raw B-scan OCT images along each of the four meridians (superior, inferior, temporal, and nasal) and marked the scleral spur as a reference point to enable the computation of the AST at intervals of 1 mm. All the images were analyzed by manual measurement to determine the AST, which was estimated from the scleral spur to a distance of 6 mm (*n* = 93) along the four meridians.

On the B-scan images of the anterior sclera, the outer boundary was identified as a thin hyporeflective region on the anterior part of the sclera, which is an actual presentation of episcleral blood vessels that separates the episclera and conjunctiva from the scleral tissue [11]. The inner boundary is a demarcated line between the hyper-reflective scleral tissue and the hyporeflective ciliary body tissue. Scleral thickness was measured as the distance between the outer and inner boundaries at the point of interest (Fig. 1).

The examiner marked the scleral spur (a slightly depressed region in the limbal area, facing the anterior chamber) as a reference point to enable the computation of scleral thickness at 1-mm intervals, extending peripherally from the scleral spur. Specifically, the thickness values were obtained along the direction normal to the tangents passing through the interval points located on the outer boundary for enhanced accuracy [10].The AST values were obtained up to 6 mm away from the scleral spur.

### Data analysis and statistics

This study used IBM SPSS 25.0.0 (SPSS, Inc., Chicago, IL, U.S.A.) software for all the statistical tests, and the inbuilt features of GraphPad Prism 8.0 (GraphPad Software, San Diego, United States) were used for plotting the graphs. Since the AST data were not found to depart significantly from a normal distribution (Shapiro–Wilk test: all *P* > 0.1 for the scleral thickness at all locations), parametric statistics were used throughout. Qualitative data were expressed as their frequency distributions. To examine the scleral thickness data in this population, a repeated-measures analysis of variance was used, with the measurement meridian (i.e., superior, inferior, nasal, and temporal) and measurement location respective to the scleral spur (i.e., 0, 1, 2, 3, 4, 5, or 6 mm). and refractive group (emmetropes, mild–moderate myopes, and high myopes), Bonferroni-corrected pairwise comparisons were performed for any significant main effects and interactions, including the continuous variables of age and SE refractive error as covariates. Additionally, we used a multiple linear regression analysis to examine age, AL, and scleral thickness measurements (i.e., the average thickness across all locations and meridians). A Student’s *t*-test was used to test differences in ocular biometry between male and female. The reliability of the AST measurements was assessed by estimating intraclass correlation coefficients (ICCs). A value of *P* < 0.05 was considered statistically significant.

## Results

This study recruited 102 Chinese participants without any ocular or systemic conditions that could influence the refractive errors of their eyes who visited Nanchang University Affiliated Eye Hospital between January 2021 and December 2021. Five participants were excluded due to unreliable OCT images, and 4 were excluded due to age. Finally, 93 participants (i.e., 93 eyes) were included in the study, including 37 males and 56 females, with a mean age of 30.2 ± 8.8 years (age range: 20 ~ 50 years). The AL was 26.51 ± 2.654 mm (range: 22.50 ~ 33.0 mm), the spherical equivalent (SE) was − 7.20 ± 6.5 D (range: +0.50 ~ 27.5 D), and the Km was 43.80 ± 1.86 D (range: 40.52 ~ 45.98 D). Based on the SE refractive error, patients were assigned into three groups: emmetropes (mean age: 29.9 ± 8.1 years), mild and moderate myopes (mean age: 28.1 ± 7.6 years), and high myopes (mean age: 32.2 ± 9.9 years).

The reproducibility of the AST measurements was excellent for intra-observer (ICC ≥ 0.963), and the AL was significantly negatively correlated with either the averaged peripheral scleral thickness (i.e., an average scleral thickness of ≤ 6 mm at peripheral locations from the limbus, superior, inferior, temporal, and nasal areas) over the 4 meridians or at 0, 1, 2, 3, 4, 5, and 6 mm per meridian (− 0.511 ≤ *r* ≤ − 0.228, *P* < 0.05). Although there was no significant correlation between age and inferior meridian (*r* = 0.113, *P* = 0.314), age was positively correlated with the average AST of the superior, temporal, and nasal diameters (the *r* ranged between 0.452 and 0.552, *P* < 0.05) (Table 2).

**Table 2:** Age- and sex- adjusted partial correlation analysis between anterior sclera thickness and axial length in myopic eyes

	superior		inferior		temporal		nasal	
	*r*	*p*	*r*	*p*	*R*	*7*	*r*	*p*
Associations with axial length								
AST(0)mm	-0.247	0.019	-0.348	0.001	-0.294	0.005	-0.276	0.008
AST(1)mm	-0.307	0.003	-0.228	0.037	-0.286	0.006	-0.304	0.003
AST(2)mm	-0.339	0.001	-0.355	0.001	-0.249	0.017	-0.438	0.000
AST(3)mm	-0.234	0.027	-0.421	0.000	**-**0.246	0.019	-0.503	0.000
AST(4)mm	-0.333	0.001	-0.326	0.002	-0.236	0.012	-0.511	0.000
AST(5)mm	-0.336	0.001	-0.296	0.006	-0.246	0.019	-0.301	0.000
AST(6)mm	-0.333	0.001	-0.238	0.030	-0.293	0.005	-0.338	0.000
Average AST	-0.398	0.000	-0.379	0.000	-0.353	0.001	-0.485	0.000
Associations with age								
Average AST	0.552	0.000	0.113	0.314	0.452	0.000	0.454	0.000

AST(0), (1), (2), (3), (4), (5), and (6)mm denotes Anterior scleral thickness at 0, 1, 2, 3, 4, 5, and 6 mm, respectively from the scleral spur

There was no significant correlation between sex and the mean AST of the four meridians in Table 3 for males (mean thickness: 559 ± 50 μm) and females (mean thickness: 552 ± 38 μm).

**Table 3:** Mean Peripheral AST by Gender and 4 meridians

meridians	Superior AST (µm)	Inferior AST (µm)	Temporal AST (µm)	Nasal AST (µm)	Average AST (µm)
Male	495 ± 42	575 ± 62	603 ± 48	569 ± 67	559 ± 50
Female	489 ± 34	573 ± 59	589 ± 46	562 ± 55	552 ± 38
*T*	-0.678	-0.130	-1.816	-0.476	-0.869
*P*	0.499	0.897	0.057	0.635	0.387

Values are shown as mean ± standard deviation (µm). Averaged anterior scleral thickness of each meridian from 0-mm to 6-mm points from the limbus

**Fig. 2:**
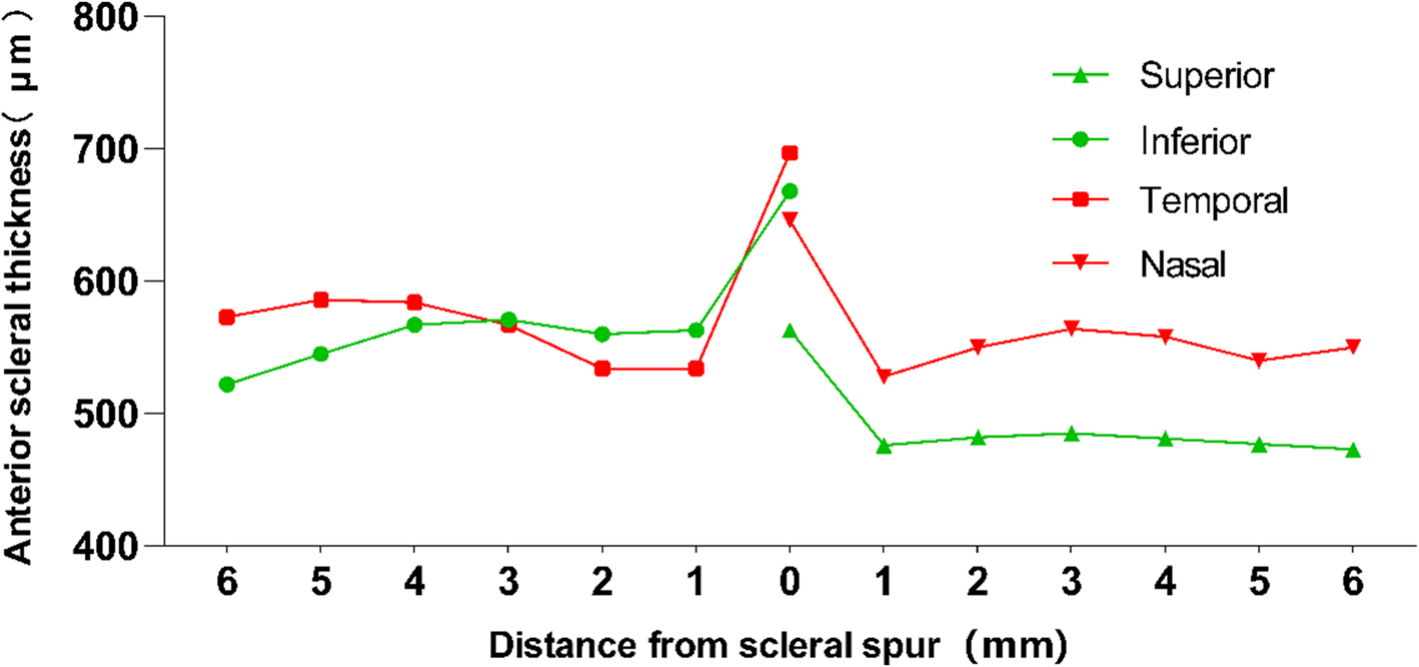
The anterior sclera thickness (AST) in the superior, inferior, temporal, and nasal meridians along different eccentricities.“0” indicates a scleral spur at the limbus. The green line with circular markers indicates the inferior meridian, and the triangular markers indicate the superior meridian. The red line with square markers indicates the temporal meridian, and the inverted triangular markers indicate the nasal meridian

The AST was thickest at the scleral spur compared with the thickness at any other location in the periphery (*P* < 0.01) along the horizontal and vertical meridians, and the differences in thickness among the meridians were as follows: temporal (697.61 ± 70.32 μm) > inferior (667.78 ± 81.22 μm) > nasal (645.49 ± 58.59 μm) > superior (560.27 ± 49.14 μm) (Table 4). The average thicknesses of the nasal (mean thickness across all locations: 564 ± 58 μm) and temporal (584 ± 50 μm) meridians were not significantly different. The thinnest was in the superior meridian compared with the inferior meridian (490 ± 36 vs. 542 ± 80 μm, *P* < 0.01) along the superoinferior meridian (Table 4; Fig. 2).

**Table 4:** AST for four meridians and distance (0 ~ 6 mm) from the Scleral Spur

Meridians	0 mm	1 mm	2 mm	3 mm	4 mm	5 mm	6 mm	Average AST
Superior	560 ± 49	474 ± 36	481 ± 40	483 ± 43	479 ± 45	478 ± 50	477 ± 52	490 ± 36
Inferior	668 ± 81	562 ± 65	560 ± 61	571 ± 66	566 ± 70	547 ± 78	542 ± 80	574 ± 60
Temporal	698 ± 70	534 ± 52	538 ± 54	567 ± 60	585 ± 67	588 ± 70	586 ± 82	584 ± 50
Nasal	645 ± 59	530 ± 59	552 ± 60	566 ± 69	560 ± 76	543 ± 93	554 ± 85	564 ± 58
Average AST	643 ± 83	524 ± 62	532 ± 62	546 ± 70	547 ± 77	539 ± 84	540 ± 85	553 ± 63

AST for each meridian superior (S), inferior (I), temporal (T), and nasal (N)) and distance (1 ~ 6 mm) from the Scleral Spur (mean ± SD (µm))

### Comparison of the difference between the mean anterior scleral thickness at 0 ~ 6 mm between the three groups and four meridians

**Fig. 3:**
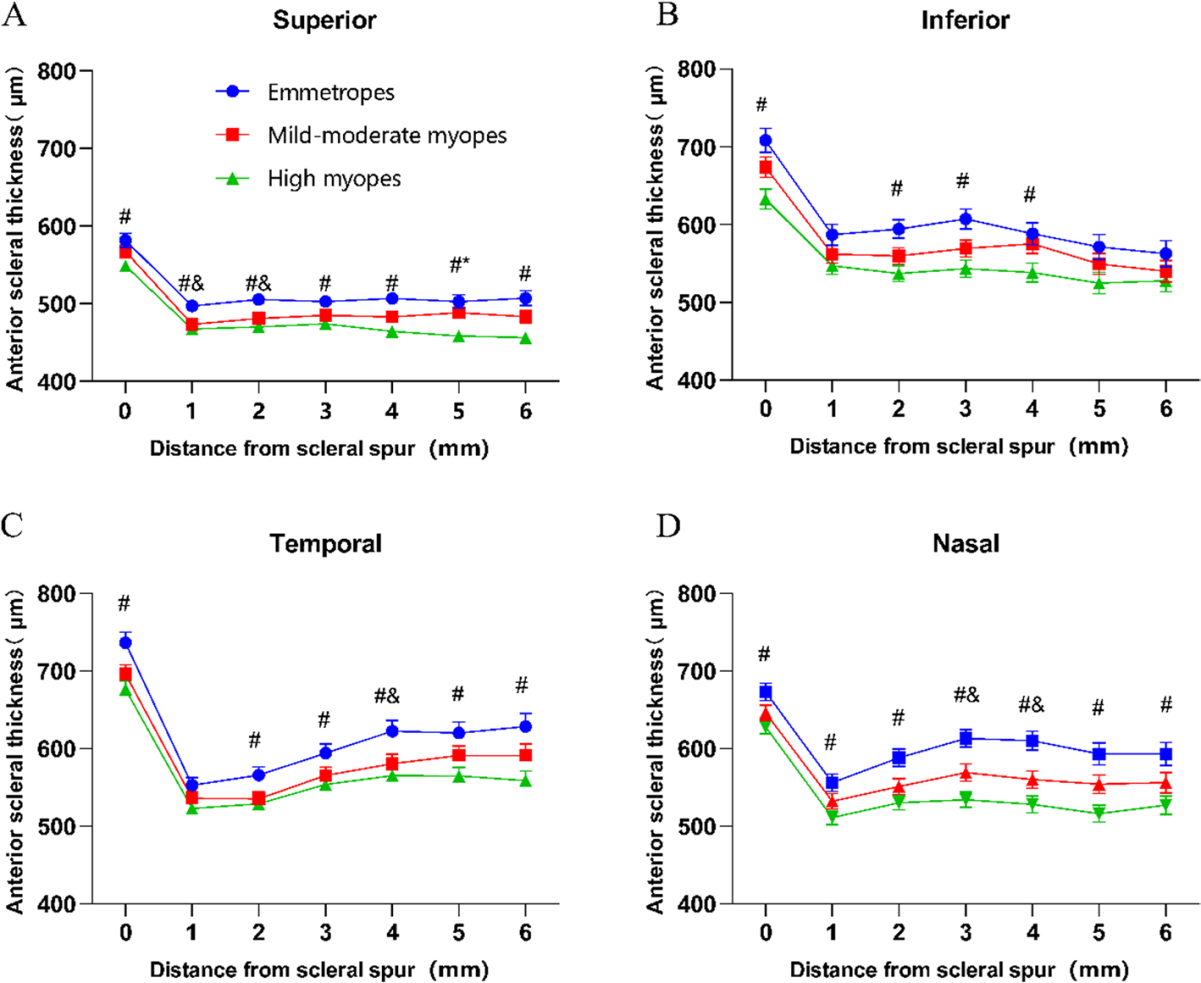
The AST (mean ± spherical equivalent) at the superior (**A**), inferior (**B**), temporal (**C**), and nasal (**D**) meridians from the scleral spur to 0 ~ 6 mm; “0” indicates the scleral spur at the limbus, and the error bar represents the standard error. Numerals 1 ~ 6 indicate the scleral position away from the scleral spur. “#” denotes a statistical significance of < 0.05 between emmetropes and high myopes. “&” denotes a statistical significance of < 0.05 between emmetropes and mild–moderate myopes. “*” denotes a statistical significance of < 0.05 between mild–moderate myopes and high myopes

The comparison of the average AST of the four diameters in the groups of emmetropes, mild-moderate myopes, and high myopes revealed a statistically significant difference in the average AST between the emmetrope and high myope groups (*F* = 6.357, *P* = 0.002; *F* = 5.853, *P* = 0.003; *F* = 5.705, *P* = 0.005; and *F* = 7.843, *P* < 0.001, respectively). Except for the inferior diameters of 1, 5, and 6 mm and the temporal diameter of 1 mm, the four diameters in the emmetrope and high myopia groups were statistically significant at 0 ~ 6 mm from the limbus (*P* < 0.05) (Fig. 3).

**Fig. 4:**
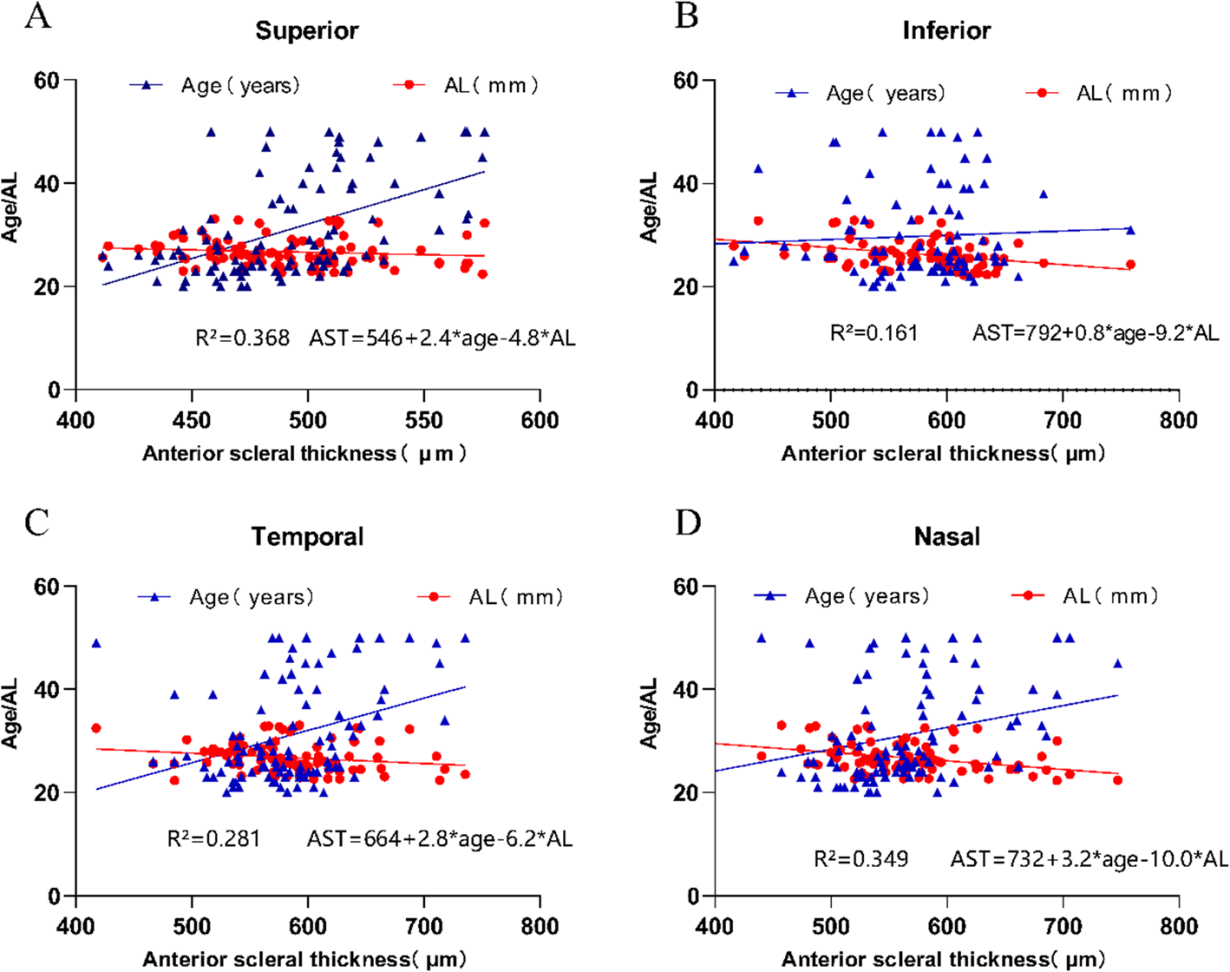
Multivariate regression fit plot of age, axial length, and mean anterior sclera thickness across the superior, inferior, temporal, and nasal meridians. Figures **A**, **B**, **C**, and **D** correspond to the superior, inferior, temporal, and nasal meridians, respectively

The multiple regression analysis (see Table 5; Fig. 4) revealed that the mean AST was significantly positively associated with age (β = 2.354, *P* < 0.05), while negatively associated with AL (β = −7.383, *P* < 0.05).

**Table 5:** Multiple regression analysis

	Unstandardized Coefficient ‘B’ (Standard Error)	t	Significance	Model R² (P-Value)
AST				
AL	-7.383 (1.319)	-5.599	< 0.001	0.376 (*P* < 0.001)
Age	2.354 (0.395)	5.952	< 0.001	

*AST *Anterior scleral thickness, *AL* Axial length

## Discussion

This study reported the AST at different eccentricities from the limbus in emmetropes and myopes using the AS-OCT spectral domain and demonstrated that significant variations in the thickness of the anterior sclera occur with age, AL, and measurement location. In all the participants, the AST was thickest at the scleral spur located in the limbal region in all the meridians; it thinned at 1-mm intervals from the scleral spur and gradually increased in thickness toward the 6-mm point from the scleral spur. Generally, the topographic variations reported in this study correspond with previous findings [8–11]. Presumably, due to the differing radii of the corneal and scleral curvature, the spur probably helps to maintain the corneal contour in an area of heightened tissue stress [12].

Age is a significant influencing factor on AST. Previous studies have shown that the structure and composition of the sclera change with age, and the cross-sectional area of scleral collagen fibrils increases with age. Moreover, the mean collagen fibril radius and intermolecular lateral spacing in the sclera also increase with age [13, 14]. The results of our study are the same as those of Read et al. [15] and Ebneter et al. [16], which reported that older age is associated with a thicker sclera. However, an examination of Chinese cadaveric eyes showed no correlation between scleral thickness and age/sex [17]. The different distances from the limbus at which the measurements were obtained and the different technology and devices used for the AST measurements may partly explain the different results.

Dhakal et al.’s [11] study did not find age to be related to AST, which may have been due to the small age range studied (average age: 24 ± 4 years). Unlike Read et al. [16] and Fernández-Vigo et al. [18] found that the AST of the nasal and temporal meridians was related to sex. Male subjects have thicker sclera, although, according to our findings, there was no significant difference between sex and the mean AST of the four meridians. Additionally, some research groups have reported no association between AST and sex [16, 17]. Therefore, there is no definite conclusion as to whether AST is correlated with sex.

In terms of the associations with AL, the results have been conflicting. Most studies did not find a significant relationship between AST and AL. Although Dhakal et al. [11] and Sung et al. [19] observed statistically significant relationships with AL at different meridians, Dhakal et al. [11] found that among all the meridians, at the scleral spur and the 1-, 2-, and 3-mm locations along the inferior meridian, the AST decreased significantly as the degree of myopia increased. Sung et al. [19] reported that increasing AL was related to anterior scleral thinning at several measurement points along the temporal, inferior, and nasal meridians. Our study also found a significant negative correlation between AL and all four meridians. The discrepancy in findings may be related to the confounding effects of age and sex. In our study, Chinese patients with a wider range of age, refractive error, and AL were recruited.

It can be speculated that the mean scleral thickness decreases with increasing AL during the dilation process; however, since the changes in scleral biomechanics are complex, the thinning of the anterior sclera may also be affected by other factors. Due to the limitation of penetration, spectral OCT, which is widely used at present, is limited when measuring posterior scleral thickness. However, since AST measurements can be obtained simply and easily, it is hoped that AST can replace the posterior sclera to evaluate the progress of myopia.

Buckhurst et al. [20] were the first to use AS-OCT to study human AST and reported thicker AST results than those found in our study. This difference is likely due to the specific definitions of scleral thickness, the inclusion of the tendons of the extraocular muscles and the episclera, and the conjunctiva from scleral tissues.

Additionally, our study found that among the four meridians, the superior meridians were the thinnest, which was consistent with the findings of previous research [8, 10, 11, 20]. The difference was that our study did not find the inferior meridians to be the thickest. One possible reason for this is when looking upwards to the fully exposed inferior sclera, the inferior scleral thickness of the distal AST is easily affected by the ciliary body and the choroid.

There are several limitations to our study that should be noted. First, this study utilized a cross-sectional approach, and follow-up longitudinal studies will be required to confirm our findings. Second, this article lacks research on the posterior sclera, and it cannot predict the sequence of AST changes and posterior scleral thickness changes; furthermore, it cannot describe the correlation between AST and the progression of myopia. Accordingly, extensive longitudinal studies and comparisons are required, and measurements of the thickness of the posterior sclera must be obtained.

In eye growth regulation, the sclera determines the size of the eye and thus, its refractive state. Increased scleral stromal remodeling can lead to the overgrowth of the eye, which can result in myopia, and an increased risk of ocular pathological complications. The sclera is a relatively safe and easily accessible drug target, and treatments targeting such changes in the sclera have the potential to reduce these complications. Future studies of a longitudinal design are necessary to establish a normative database of AST in a larger sample, which may be useful for providing reference values and assessing the degree of myopia progression.

In conclusion, this study reported the distribution of AST in emmetropic eyes and different myopic eyes. It revealed that scleral thickness varies significantly with AL and measurement location and found AST to be negatively correlated with AL and positively correlated with age. Compared with emmetropic eyes, the anterior sclera in highly myopic eyes is thinner. Myopia affects not only posterior scleral thickness but also AST. Accordingly, AST may be useful for monitoring the progression of myopia.

## Data Availability

The datasets used and/or analysed during the current study available from the corresponding author on reasonable request.

## Abbreviations

OCT: Optical Coherence Tomography
AS-OCT: Anterior Segment Optical Coherence Tomography
AST: Anterior Scleral Thickness
AL: Axial Length
SE: Spherical Equivalent
D: Diopter
Km: Mean meridian keratometry

## References

[1] Holden BA, Fricke TR, Wilson DA (2016). Global prevalence of myopia and high myopia and temporal Trends from 2000 through 2050. Ophthalmology.

[2] Howlett MH, Mcfadden SA (2007). Emmetropization and schematic eye models in developing pigmented guinea pigs. Vision Res.

[3] Li J, Lu J, Chen G, Li D (2022). Andrographolide protects retinal ganglion cells in rats with glaucoma by regulating the bcl-2/bax/ caspase-3 signaling pathway. World J Tradit Chin Med.

[4] Wallman J, Winawer J (2004). Homeostasis of eye growth and the question of myopia. Neuron.

[5] Siegwart JJ, Norton TT (2011). Perspective: how might emmetropization and genetic factors produce myopia in normal eyes?. Optom Vis Sci.

[6] Ouyang X, Han Y, Xie Y (2019). The collagen metabolism affects the scleral mechanical properties in the different processes of scleral remodeling. Biomed Pharmacother.

[7] Mcbrien NA, Jobling AI, Gentle A (2009). Biomechanics of the sclera in myopia: extracellular and cellular factors. Optom Vis Sci.

[8] Buckhurst HD, Gilmartin B, Cubbidge RP (2015). Measurement of Scleral Thickness in Humans Using Anterior Segment Optical Coherent Tomography. PLoS One.

[9] Vurgese S, Panda-Jonas S, Jonas JB (2012). Scleral thickness in human eyes. PLoS One.

[10] Pekel G, Yağcı R, Acer S, Ongun GT, Çetin EN, Simavlı H (2015). Comparison of corneal layers and anterior sclera in emmetropic and myopic eyes. Cornea.

[11] Dhakal R, Vupparaboina KK, Verkicharla PK (2020). Anterior Sclera Undergoes Thinning with Increasing Degree of Myopia. Investig Ophthalmol Vis Sci.

[12] Boote C, Hayes S, Young RD (2009). Ultrastructural changes in the retinopathy, globe enlarged (rge) chick cornea. J Struct Biol.

[13] Malik NS, Moss SJ, Ahmed N, Furth AJ, Wall RS, Meek KM (1992). Ageing of the human corneal stroma: structural and biochemical changes. Biochim Biophys Acta.

[14] Daxer A, Misof K, Grabner B, Ettl A, Fratzl P (1998). Collagen fibrils in the human corneal stroma:structure and ageing. Invest Ophthalmol Visual Sci.

[15] Read SA, Alonso-Caneiro D, Vincent SJ (2016). Anterior eye tissue morphology: scleral and conjunctival thickness in children and young adults. Sci Rep.

[16] Ebneter A, Häner NU, Zinkernagel MS (2015). Metrics of the normal anterior sclera: imaging with optical coherence tomography. Graefes Arch Clin Exp Ophthalmol.

[17] Shen L, You Q, Xu X (2015). Scleral thickness in Chinese eyes. Investig Ophthalmol Vis Sci.

[18] Fernández-Vigo JI, Shi H, Burgos-Blasco B (2022). Anterior scleral thickness dimensions by swept-source optical coherence tomography. Clin Exp Optom.

[19] Sung MS, Ji YS, Moon HS (2021). Anterior scleral thickness in myopic eyes and its association with ocular parameters. Ophthalmic Res.

[20] Norman RE, Flanagan JG, Rausch SM (2010). Dimensions of the human sclera: thickness measurement and regional changes with axial length. Exp Eye Res.

